# Multitrophic interactions among Western Corn Rootworm, *Glomus intraradices* and microbial communities in the rhizosphere and endorhiza of maize

**DOI:** 10.3389/fmicb.2013.00357

**Published:** 2013-12-11

**Authors:** Flavia Dematheis, Benedikt Kurtz, Stefan Vidal, Kornelia Smalla

**Affiliations:** ^1^Julius Kühn-Institut - Federal Research Centre for Cultivated Plants, Institute for Epidemiology and Pathogen DiagnosticsBraunschweig, Germany; ^2^Department of Crop Science, Agricultural Entomology, Georg-August University GöttingenGöttingen, Germany

**Keywords:** Western Corn Rootworm, *Glomus intraradices*, rhizosphere, endorhiza, ITS, 16S rRNA gene, denaturing gradient gel electrophoresis, 18S rRNA gene restriction fragment length polymorphism

## Abstract

The complex interactions among the maize pest Western Corn Rootworm (WCR), *Glomus intraradices* (*GI*—recently renamed *Rhizophagus intraradices*) and the microbial communities in both rhizosphere and endorhiza of maize have been investigated in view of new pest control strategies. In a greenhouse experiment, different maize treatments were established: C (control plants), W (plants inoculated with WCR), G (plants inoculated with *GI*), GW (plants inoculated with *GI* and WCR). After 20 days of WCR root feeding, larval fitness was measured. Dominant arbuscular mycorrhizal fungi (AMF) in soil and maize endorhiza were analyzed by cloning of 18S rRNA gene fragments of AMF, restriction fragment length polymorphism and sequencing. Bacterial and fungal communities in the rhizosphere and endorhiza were investigated by denaturing gradient gel electrophoresis of 16S rRNA gene and ITS fragments, PCR amplified from total community DNA, respectively. *GI* reduced significantly WCR larval development and affected the naturally occurring endorhiza AMF and bacteria. WCR root feeding influenced the endorhiza bacteria as well. *GI* can be used in integrated pest management programs, rendering WCR larvae more susceptible to predation by natural enemies. The mechanisms behind the interaction between *GI* and WCR remain unknown. However, our data suggested that *GI* might act indirectly via plant-mediated mechanisms influencing the endorhiza microbial communities.

## Introduction

The Western Corn Rootworm (WCR), *Diabrotica virgifera virgifera* LeConte, is an invasive maize pest in North America and in Europe (Wesseler and Fall, 2010). WCR larvae feed on maize root tissues causing bent stalks (goose necking) and lodging. Economic losses are mainly due to difficulties in mechanical harvesting of injured maize plants.

For large-scale farming operations the main options in controlling the WCR include chemical control, the use of transgenic plants and crop rotation. Unfortunately, the repeated use of pesticides can provide high selective pressure, which can lead to chemical resistance in the WCR populations, resulting in poor control of the pest, increasing insecticide application rate and control costs (Meinke et al., 1998; Siegfried et al., 2004). With the crop biotechnology *Diabrotica*-resistant transgenic maize expressing the *cry(3Bb1)* gene from the bacterium *Bacillus thuringiensis kumamotoensis* (Bt maize) has been introduced already in 2003 (Vaughn et al., 2005; Hellmich et al., 2008). The concentration of *cry(3Bb1)* expressed in Bt maize is not considered a high dose for WCR (Al-Deeb and Wilde, 2005; Oyediran et al., 2007), and resistance was reported to build up within three generations of selection on Bt maize in greenhouse experiments (Meihls et al., 2008; Gassmann et al., 2011). Another strategy, widely used in the past in the United States (U.S.) for managing the WCRs is the crop rotation. Corn rotated annually with soybeans was, in fact, not susceptible to rootworm larval damage as WCR adults laid eggs exclusively in cornfields and larvae hatched in soybeans starved to death. Unexpectedly, the intensive annual rotation of corn with soybeans caused in the U.S. the selection of a WCR variant with reduced egg-laying fidelity to maize field (Onstad et al., 2001; Levine et al., 2002; Spencer et al., 2009). As a consequence of rotation resistance, farmers have experienced, since 1995, economic losses caused by WCR larval injury to first-year maize. In Europe, where only the WCR wild type is present, the best management option remains, up to now, the crop rotation. However, it is clear that due to the development in the WCR populations of resistances against the main WCR pest control options described above, new and long-term resistance management strategies need to be developed. An improved knowledge of the ecology of this soil-dwelling insect and its multitrophic interactions in the rhizosphere and endorhiza are important prerequisites to achieve this goal.

The rhizosphere and endorhiza are dynamic environments in which plant, fungi, bacteria, viruses, nematodes and herbivore insects interact with each other influencing the agro-ecosystem functionality, and thus the sustainability of the crop production (Weller and Thomashow, 1994; Berg and Smalla, 2009). Beneficial rhizosphere microorganisms promote plant growth and health by nutrient solubilization, nitrogen fixation and plant hormone production (Hayat et al., 2010). Microbial endophytes influence plant fitness as well, affecting plant-microbe-arthropod interactions (Finkes et al., 2006; Rudgers et al., 2007). Within the endophytes, the arbuscular mycorrhizal fungi (AMF) are well known to improve plant survival in harsh environments by enhancing several plant functions (Newsham et al., 1995; Smith and Read, 2008) including drought resistance (Davies et al., 2002), tolerance to heavy metal contaminations (Gildon and Tinker, 1983), protection against pathogens through microbial antagonism and increased plant defensive capacity (Newsham et al., 1995). Furthermore, AMF are prominent through their well-established ability to affect insect-herbivore-plant interactions (Gehring and Bennett, 2009). Several reports showed that AMF can affect the behavior, development and insect performance (Gange et al., 1994; Wardle, 2002; Davet, 2004; Bezemer and van Dam, 2005; Hartley and Gange, 2009; Koricheva et al., 2009), either changing the nutritional status of the plant or triggering plant defense responses (Goverde et al., 2000; Nishida et al., 2010). Bennett and Bever (2007) showed that plant feeders tend to be negatively or positively influenced by the AMF species which the plant is associated with. In particular, the mycorrhizal fungus *Glomus* white does not alter the response of the narrow-leaved plantain (*Plantago lanceolata*) to the specialist lepidopteran herbivore, *Junonia coenia*; the plant association with the AMF *Archaeospora trappei* leads to tolerance to herbivore in the form of an increased plant growth rate; the association with the fungus *Scutellospora calospora* reduces plant tolerance to the herbivores. It must be noticed that, due to monitoring difficulties, belowground herbivore insects have been seldom examined. However, Gange et al. (1994) showed the effect of the AMF, *Glomus mosseae*, on the reduction of black vine weevil (*Otiorhynchus sulcatus Fabricius*) larval growth.

It has been shown that AMF may influence directly or indirectly the activity and the community structure of the rhizosphere- and root-associated microorganisms either through the release of hyphal compounds or through changes in the plant root exudation patterns (Marschner and Baumann, 2003; Wamberg et al., 2003; reviewed by Jones et al., 2004; Offre et al., 2007). The microbial community assembly can be affected also by belowground insect attackers (Denton et al., 1998; Grayston et al., 2001; Dawson et al., 2004; Currie et al., 2006). Upon insect attacks, changes in the plant transcriptome, in the production of volatiles or root exudates have been often detected (Köllner et al., 2008; Dicke et al., 2009). Root feeding effects of WCR larvae on the bacterial and fungal community composition in the maize rhizosphere were recently observed (Dematheis et al., 2012). However, effects of WCR larval feeding on the indigenous microbial communities inhabiting the maize endorhiza remained up to now unexplored. In addition, no studies on the effect of *Glomus intraradices* (GI), recently renamed *Rhizophagus intraradices* (Schüßler and Walker, 2010), on WCR larval fitness and on both rhizosphere and endorhiza microbes of maize have been reported yet.

The present study aimed to investigate the multitrophic interaction among WCR, *GI* and the microbial communities in the rhizosphere and endorhiza of maize. We specifically addressed the following questions: (1) Does *GI* mycorrhization of maize roots affect the WCR larval fitness measured as larval number/survival, developmental stage and root feeding? (2) Does *GI* mycorrhization affect the composition of microbial populations in the rhizosphere and endorhiza of maize? (3) Does the feeding of WCR larvae alter the microbial communities in the endorhiza and rhizosphere of mycorrhized and unmycorrhized maize plants?

In the present study AMF, total fungal and bacterial communities were investigated. AMF communities naturally occurring in the soil and colonizing the maize endorhiza were studied by PCR-RFLP analysis and sequencing of AMF-specific 18S rRNA gene fragments, PCR amplified from total community (TC) DNA. The total fungal and bacterial communities in both rhizosphere and endorhiza of maize were analyzed by means of denaturing gradient gel electrophoresis (DGGE) of ITS and 16S rRNA gene fragments, PCR amplified from TC-DNA.

## Materials and methods

### Experimental setup

A greenhouse experiment was performed under quarantine conditions. The maize variety used in this study was KWS13, an early maturing Northern European flint x dent maize breeding line developed by the seed company KWS (Einbeck, Germany). Maize seeds were sterilized according to Benziri et al. (1994) and pre-germinated at room temperature in Petri dishes containing sterile wet filter paper. The seedlings were pre-grown singly in pots (13 cm diameter) containing Haplic Chernozem inoculated or not with *GI* for 6 weeks. The maize growing conditions were 40% relative humidity, 24°C mean temperature and 16 h of additional illumination with sodium lamps (400W, HS2000, Hortilux Schréder, Monster, The Netherlands). Plants were placed into the same tray that was moved twice a week in the greenhouse to randomize the growing conditions. Every 14 days of growth, each plant was fertilized with 20 μl 0.2% Wuxal top N (Manna, Düsseldorf, Germany) by watering.

After six weeks of plant growth (plant growth stage V7) four plant replicates per treatments with and without *GI* were harvested in order to quantify by real-time PCR (qPCR) the *GI*-root colonization. The remaining plants were used to assess the following treatments: C (control plant grown in Haplic Chernozem), W (maize plants inoculated with ~200 eggs), G (maize plants mycorrhized by *GI*) and GW (maize plants mycorrhized by *GI* and inoculated with ~200 non-diapausing WCR eggs). Because of logistic constraints only four independent replicates (one replicate = one plant) per treatment were established. Three weeks later (plant growth stage VT) the larvae were collected from the treatments W and GW to evaluate the total number of larvae per plant and the development of the larval instars (L1, L2, and L3). In parallel, the plants were harvested, and the fresh weight of the roots was recorded. After the rhizosphere isolation the roots were surface sterilized. TC-DNA was extracted from soil, rhizosphere and surface sterilized roots in order to determine (a) the 18S/ITS rRNA gene copy numbers of *GI* in the roots by qPCR; (b) the AMF community structure in soil and roots by cloning of 18S rRNA gene fragments of AMF, restriction fragment length polymorphism and sequencing, and (c) the bacterial and fungal community assembly in the rhizosphere and endorhiza by DGGE analysis of PCR-amplified 16S rRNA gene and ITS fragments.

### Soil type and sampling method

The soil used in this study was Haplic Chernozem, collected in 2008 nearby Göttingen (geographic coordinates, 51°30′29.44 N and 9°55′38.26 E). 400 kg were taken from four different spots, five meters apart from each other, along a transect to a depth of 25 cm. In order to avoid any alteration of the microbial content, the soil samples were immediately transported to the laboratory and homogenized by a soil crusher machine (Unifix 300, Möschle, Ortenberg, Germany) and sieved through a 10 mm mesh to remove stones and plant residues. Fresh soil was used for the experiment described here.

### *Glomus intraradices* inoculum and soil application

The arbuscular mycorrhizal *Glomus intraradices* (Glomeromycota) was provided by Dr. Henning von Alten (Isolate n° 501, Institute of Plant Disease and Plant Protection, University of Hannover, Germany) as expanded clay material contains a high level of *GI* spores. The inoculum was mixed as 5% of the total volume of soil estimated for the whole experiment (Dehne and Backhaus, 1986).

### WCR egg inoculum and application

Non-diapausing WCR eggs were provided by USDA-ARS (Northern Grain Insect Research Laboratory, Brookings, USA) and stored at 8°C until their use. In order to stimulate the larval development, the eggs were incubated at 26°C, 60% relative humidity in dark conditions for 12 days and checked for visible larvae presence using a dissecting microscope. Afterwards the eggs were washed in a sieve (Ø 250 μm) and the collected eggs were suspended in 0.15% agar solution. 0.5 ml of egg suspension were applied on a sterile humid filter paper and incubated at the same conditions as described for larval development, and checked daily to assess the hatch time (HT) and the hatch rate (HR). HT and HR mean values were two days and 72%, respectively. Approx. 200 eggs were applied into the soil, at 5 cm depth close to the stem of the plants for the establishment of the treatments W and GW.

### WCR larval extraction from the soil, larval development analysis, root feeding evaluation and statistics

Larvae were extracted from the soil of plants inoculated with WCR eggs (treatments W and GW) using a high gradient Kempson extraction system (Kempson et al., 1968). The larvae extracted from each plant were counted and classified into larval stages (L1, L2, and L3) by measuring head capsule width as described by Hammack et al. (2003). The WCR root feeding was evaluated based on the root fresh weight of four plant replicates for each treatment.

The root weight values and total numbers of larvae per plant were analyzed with One-Way ANOVA combined with Tukey's HSD test to evaluate statistical differences among treatments. The analysis of the composition of larval stages was performed using a Tukey's HSD test under a generalized linear model via a logistic function for binomial data. The program used was R add-on package multicomp.

### Total community (TC) DNA extraction from rhizosphere and root samples

Maize plants at the growth stages V7 and VT were taken out from the soil and shaken vigorously. The soil tightly adhering to the roots was considered as rhizosphere and collected using a Stomacher blender (Stomacher 400, Seward, England) as described by Costa et al. (2006). The microbial pellet was obtained from the cell suspensions by centrifugation at 10,000 g at 4°C for 30 min. The microbial pellet of each root was homogenized with a spatula and 0.5 g were used for the TC-DNA extraction.

Fresh root material was prewashed under running tap water and surface sterilized as described by Götz et al. (2006). Afterwards, each root was cut into 1 cm-segments and mixed to randomize the selection of different root areas. 0.4 g of root pieces per plant were used for the TC-DNA extraction.

The TC-DNA was extracted from 0.5 g of rhizosphere pellet and from 0.4 g of surface sterilized root pieces using the FastDNA SPIN Kit for Soil (Q-Biogene, Carlsbad, CA, USA) according to the manufacturer's protocol. The treatment of the root material required the following additional initial step, root fragments were placed into bead tubes containing a mixture of ceramic and silica particles (included in the kit), frozen by immersion into liquid nitrogen and subsequently processed twice for 1 min at speed 5.5 ms^−1^ in a FastPrep bead beating system (Bio-101, Vista, CA, USA). All TC-DNA samples were purified with the GENECLEAN Spin Kit (Q-Biogene, Heidelberg, Germany) according to the manufacturer's protocol. DNA concentrations were estimated visually by 0.8% agarose gel electrophoresis using the quantitative marker High DNA Mass Ladder (*Invitrogen*). TC-DNA from both rhizosphere and root samples were diluted in MilliQ sterilized water to obtain ca. 20 ng/μl for use as a PCR template.

### Detection and quantification of GI by quantitative real-time pcr (qPCR)

The abundance of *GI* in the maize roots of all treatments was determined by means of qPCR using the primer pair VC-F/VC-R targeting in a specific manner the ITS1+18SrRNA gene fragments of the mycorrhizal fungus (Alkan et al., 2006). The qPCR was carried out in the CFX96 Real Time PCR System (Biorad, Hercules, California). The reaction mixture and cycling program were performed as described by Alkan et al. (2006) with few modifications, 25 μl aliquot of reaction mixture contained 1 μl DNA template and 2X SYBR Green qPCR Master Mix (Fermentas, St. Leon-Rot, Germany).

The qPCR was calibrated with the cloned ITS1+18SrRNA fragment of the *GI* strain used in this study. From the standard calibration curves, the amount of *GI* in 1 g of plant root was calculated.

The standard for the qPCR was prepared as follows: TC-DNA of roots colonized by *GI* was amplified as described above by Alkan et al. (2006). Amplicons, 110 bp length, were ligated in the pGEM-T vector system (Promega) and transformed into *Escherichia coli* (JM109 Competent Cells, Promega) according to the manufacturer's instructions. Positive transformants were re-amplified in a Tgradient thermal cycler (Biometra, Göttingen, Germany) with the primers SP6 and T7, purified with the “MinElute PCR purification Kit” (Qiagen GmbH, Hilden, Germany) and sequenced. The BLAST analysis of DNA sequences at NCBI site showed 100% identity with *GI* (accession no. JN83667-JN836670). The PCR products from single clones amplified with SP6 and T7 were quantified with the *NanoDrop* Spectrophotometer ND-1000 (Peqlab, Erlangen, Germany) and serial dilutions 10^−4^ to 10^−10^ were used as a standard for the detection and quantification of *GI* in the root samples.

### Cloning of 18s rRNA gene fragments of AMF, restriction fragment length polymorphism (RFLP) and sequencing

To investigate the AMF communities, the partial 18S rRNA gene fragments (550 bp) were amplified from TC-DNA extracted from a composite soil sample and four root samples from each treatment. The PCR was performed with the primer pair NS31/AM1 according to Vallino et al. (2006) with the following modifications, no bovine serum albumin (BSA) was added to the PCR reaction mixture and 2 U of Taq DNA polymerase (AmpliTaqGold with GeneAmp, Applied Biosystems, USA) and 10 pmol of each primer were used. Moreover, the PCR extension temperature was increased to 62°C. PCR modifications were made to optimize the AMF amplification in the soil. Due to a multiple pattern obtained from the soil sample, 550 bp length amplicons were cut out from the agarose gel and purified by “QIAEXII gel extraction kit” (Qiagen GmbH, Hilden, Germany).

Amplicons of 550 bp length from soil and roots were ligated in the pGEM-T vector system (Promega) and transformed into *Escherichia coli* (JM109 Competent Cells, Promega) according to the manufacturer's instructions. Positive transformants were amplified with the primer pair NS31/AM1 to select the clones carrying the insert with the right size. The PCR conditions were optimized for the cloned target sequence as follows, 95°C for 10 min, 30 cycles at 94°C for 35 s, 63°C for 35 s, 72°C for 45 s, and final step at 72°C for 10 min. Positive clones (180 clones obtained from a soil composite sample and 140–155 clones obtained from root samples for each treatment) were tested for RFLP type by independent digestion with the enzymes *HinfI* and *Hin1II* (Fermentas), as recommended by the manufacturer and analyzed on 3% agarose gel electrophoresis. For an appropriate identification of the size of restricted fragments, the Molecular weight marker IX (Boehringer Mannheim GmbH, Germany) was used as a standard. Each clone was identified as RFLP type according to Vallino et al. (2006). Representative clones for each RFLP type were re-amplified with the primers SP6 and T7, purified with the “MinElute PCR purification Kit” (Qiagen GmbH, Hilden, Germany) and sequenced. The DNA sequences were analyzed by BLAST-n program at the NCBI site for multiple sequence alignment.

### PCR amplification of the internal transcribed spacer (ITS) regions and 16s rRNA gene fragments for DGGE fingerprinting

ITS fragments of the fungal communities in the endorhiza and rhizosphere of maize were amplified from TC-DNA extracted from plants of the treatments C, W, G, and GW. The ITS amplification was performed using a nested PCR approach with the primer pair ITS1F/ITS 4 and ITS 2/ITS1F-GC according to Weinert et al. (2009).

The 16S rRNA gene fragments of complex bacterial populations contained in the same set of samples were amplified by direct PCR performed with the primer pair F984GC/R1378 (Heuer et al., 1997). PCR conditions were applied as described by Costa et al. (2006).

### Denaturing gradient gel electrophoresis (DGGE) and data analysis

The DGGE analyses of the fungal and bacterial communities were carried out in the PhorU2 machine (Ingeny, Goes, The Netherlands). DGGE gels were prepared as described by Weinert et al. (2009). Gels were silver-stained and air-dried according to Heuer et al. (2001). Digitalized DGGE gel images were analyzed with the software package GELCOMPAR II program, version 4.5 (Applied Math, Kortrijk, Belgium) as described by Rademaker et al. (1999). Background was subtracted and lanes were normalized as described by Gomes et al. (2003). Cluster analysis based on the Pearson correlation coefficient (UPGMA) was performed to evaluate the percentage of similarities among samples.

Pairwise statistical analysis (Permutation test) was applied on the values of the similarity matrix according to Kropf et al. (2004) to evaluate if the differences (*D*-values) observed were statistically supported. *P*-values and *D*-values were always reported.

### Identification of specific endorhiza fungi by DGGE fingerprints of ITS fragments band sequencing

Four bands of the fungal DGGE fingerprints of ITS fragments bands which occur exclusively in the roots of plants treated with *GI* (treatments G and GW) were excised from the acrylamide gel. DNA was eluted during overnight incubation of the gel slices at 4°C in sterile TE buffer, pH 8. After centrifugation at 11,000× *g* for 60 s, the supernatant was transferred to a new tube and 1 μl of it was used as a template in the second PCR amplification described for DGGE fingerprints of ITS fragments analysis, except for the use of primers without GC clamp (ITS1F/ITS2). PCR products were ligated into the pGEM-T vector system (Promega) and transformed into *Escherichia coli* (JM109 Competent Cells, Promega) according to the manufacturer's instructions. Positive clones were re-amplified with the primers ITS1F-GC/ITS2 and the electrophoretic mobility of the cloned fragments was checked by DGGE gel. To identify different ribotypes co-migrating on acrylamide gel, four to five clones per excised DGGE band were sequenced. The DNA sequences were analyzed with BLAST-n program at NCBI site for multiple sequence alignments with sequences available in the database.

### Nucleotide sequence accession numbers

Nucleotide sequences determined in this study were deposited in the GenBank database under the accession numbers JN836634-JN836670.

## Results

### *GI* detection and quantification in maize roots

In order to assess *GI* abundance in the endorhiza of maize before WCR egg inoculation, a qPCR was performed on TC-DNA extracted from roots of maize at the growth stage V7 with and without *GI* inoculant (C, G). The qPCR revealed a specific *GI*-signal exclusively in the roots of plants grown in the soil inoculated with *GI* (G) with a mean of 9.5 × 10^5^ copy numbers of 18S/ITS fragments per g root.

The roots of maize at the growth stage VT of each treatment (C, W, G, and GW) were analyzed by qPCR as well, in order to study the treatment effect on *GI* root colonization. A specific qPCR signal was detected only in the roots of plants grown in soil inoculated with *GI* in presence and in absence of WCR larvae (G, GW). The *GI* mean value was about 1.8 × 10^6^ (s.d. 0.2) and 2 × 10^6^ (s.d. 0.3) copies of 18S/ITS fragments per g root in the treatments G and GW, respectively. No significant differences were observed between these treatments (*P* = 0.8) indicating that WCR larval feeding did not influence the abundance of *GI* in the roots. Differences in the *GI* abundance were observed instead between plants at the growth stages V7 and VT (*P* < 0.05), indicating that the mycorrhization increased during the 9 weeks of plant growth.

### GI-soil inoculation affects larval development

In order to evaluate the effect of *GI* on the root biomass and on the WCR root feeding, the root fresh weight of plants at the growth stage VT from the treatments C, W, G, and GW was determined. Significant differences of the root fresh weight between the treatments with and without larvae (*P* < 0.01) indicated a clear larval effect on the root biomass with ~20% reduction of the root weight for the treatments W and GW. No significant differences of root biomass were observed between the treatments with and without *GI* soil inoculation (C/G and W/GW), indicating that *GI* mycorrhization did not improve the belowground plant development and did not affect the root larval feeding.

**Figure 1:**
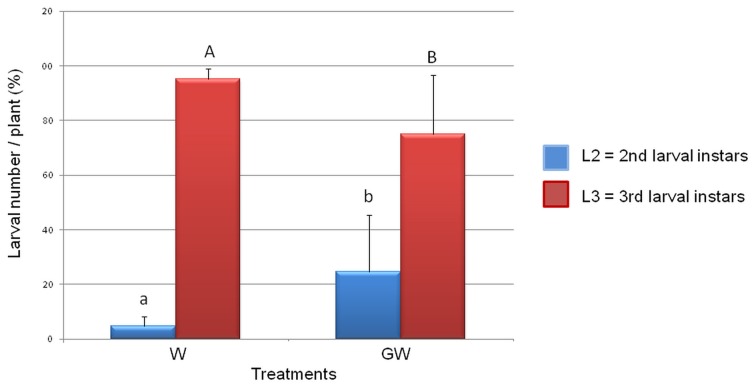
**Effect of *Glomus intraradices* on WCR larval development**. The number of 3rd larval instars (L3) was significantly lower in the *Glomus*-treated plants (treatment GW) than in untreated control plants (treatment W). Four biological replicates per treatment were used. The error bars represent standard deviations. Lowercase letters above columns indicate significance differences between the number of L2 larval instars, while uppercase letters indicate significance differences between L3 larval numbers (*P* = 2e^−16^).

The numbers of WCR larvae determined for the treatments W and GW did not significantly differ from each other indicating that *GI* mycorrhization did not affect the viability of the WCR eggs or the larval survival. However, the analysis of the larval instars composition in the treatments W and GW revealed a significant reduction of the WCR larval development in presence of the *GI* (Figure 1) with the relative number of 3rd larval instars being significantly lower in the GW than in the W treatment (*P* = 2e^−16^).

### AMF composition in soil and root samples

In order to assess (i) the AMF community structure in the soil, (ii) the AMF populations naturally occurring in the maize roots and (iii) the effect of both *GI*-soil inoculation and WCR larval feeding on the endorhiza AMF communities, a PCR-RFLP analysis was performed on the TC-DNA extracted from one composite soil sample and from four root samples per treatment (C, W, G and GW). The PCR-RFLP analysis of 180 clones carrying the 18S rRNA gene fragments of AMF obtained from the soil sample, revealed five different RFLP patterns including RFLP types 1, 2, 3, 6, 8, and several (ca. 32%) unclassified RFLP types. Among the unclassified RFLP profiles, one occurred more often and was termed as RFLP X. The dominant AMF in the soil belonged to the RFLP types 8 and 1. The percentage of clones carrying 18S rRNA gene fragments of AMF on the total number of clones investigated by means of RFLP method are reported in Table 1. The RFLP analyses of 140–155 cloned 18S rRNA gene fragments obtained from root samples per treatment revealed that the AMF colonizing the maize roots from the treatments C and W belonged to the RFLP types 2, 3, 6, and 8. In these roots, the RFLP types 8 and 3 were dominant. Differently, in the roots of plants from the treatments G and GW the RFLP analysis showed a significant reduction of the AMF evenness and almost all clones were assigned to the RFLP type 11. Cloned 18S rRNA gene fragments representative of each RFLP type were sequenced and virtually digested with the enzymes *Hinf1* and *Hin1II* in order to obtain clear information about the restriction fragment lengths characterizing each RFLP type. Database searches of 18S rRNA gene sequences representative of each RFLP type allowed the identification of different AMF species from the genera *Scutellospora* (RFLP type 6) and *Glomus* (RFLP types 1, 2, 3, 8, 11, and X). In Table 2 the RFLP types found in both soil (Haplic Chernozem) and plant roots, the source of isolation, the corresponding accession number, the species with highest sequence identity found in the GenBank, and the exact coordinates and restriction fragment lengths are reported.

**Table 1:** **RFLP types and their relative abundance in Haplic Chernozem and in root samples from the treatments C, W, G, and GW grown in the same soil type**.

**RFLP type**	**Relative abundance of RFLP types in soil and maize roots**				
	**Soil**	**Treatment C**	**Treatment W**	**Treatment G**	**Treatment GW**
RFLP 1	14.4	0	0	0	0
RFLP 2	10	5.8	3.5	0	0
RFLP 3	2.2	18.7	25.3	0	0
RFLP 6	1.1	2.5	1.4	0	0
RFLP 8	40	62	60	7.5	5
RFLP 11	0	0	0	93	94.3
RFLP X	6.7	0	0	0	0
Unclassified RFLP profiles	25.5	11	9.8	0	0

C, maize plant grown in Haplic Chernozem, natural source of different mycorrhizal species; W, maize plants characterized by 3 weeks root feeding of WCR larvae; G, maize plants mycorrhized by GI; GW, maize plants mycorrhized by GI and characterized by 3 weeks root feeding of WCR larvae. The relative abundance of the RFLP types found in soil and roots was calculated as percentage of clones carrying the insert of a certain RFLP type on the total number of clones digested with HinfI and Hin1II per soil or plant treatment.

**Table 2:** **RFLP types found in the soil Haplic Chernozem and in plant roots from the treatments C (control plants), W (maize plants characterized by 3 weeks root feeding of WCR larvae), G (maize plants mycorrhized by *GI*) and GW (maize plants mycorrhized by *GI* and characterized by 3 weeks root feeding of WCR larvae); accession numbers; sequence identity; AMF-18S rRNA gene fragment coordinates and restriction fragment lengths obtained with the enzymes *Hinf1* and *Hin1II* by virtual digestion at BioLabs web site**.

**RFLP type**	**Source**	**Access. no**	**Identity sequence (ID)**	***Hinf 1***		***Hin1II***	
				**Coordinates**	**Length (bp)**	**Coordinates**	**Length (bp)**
RFLP1	Soil	JN836649	*G. etunicatum* (99% ID)	268–552	285	1–297	297
				1–267	267	388–552	165
						298–387	90
RFLP2	Soil	JN836650	*Uncultured Glomus* (99% ID)	280–523	244	258–548	291
	Root C	JN836641		1–189	189	1–257	257
	Root W	JN836645		190–279	90		
				524–548	25		
RFLP3	Soil	JN836651	*Uncultured Glomus* (98% ID)	280–523	244	258–548	291
	Root C	JN836642		1–189	289	1–164	164
	Root W	JN836646		190–279	90	165–257	93
				524–548	25		
RFLP 6	Soil	JN836652	*Scutellospora calospora* (99% ID)	1–301	301	260–547	288
	Root C	JN836643		302–522	221	1–169	169
	Root W	JN836647		523–547	25	170–259	90
RFLP8	Soil	JN836653	*G. mosseae* (100% ID)	267–550	284	1–295	295
	Root C	JN836644		23–266	244	296–438	143
	Root W	JN836648		1–22	22	439–550	112
	Root G	JN836636-37					
	Root GW	JN836640					
RFLPX	Soil	JN836654	*G. aurantium* (99% ID)	283–550	268	260–550	291
				1–141	141	1–169	169
				142–282	141	170–259	90
RFLP 11	Root G	JN836634-35	*G. intraradices* (99% ID)	142–524	383	259–549	291
	Root GW	JN836638-39		1–141	141	117–258	142
				525–549	25	1–116	116

### Fungal communities in the endorhiza and rhizosphere of maize

**Figure 2:**
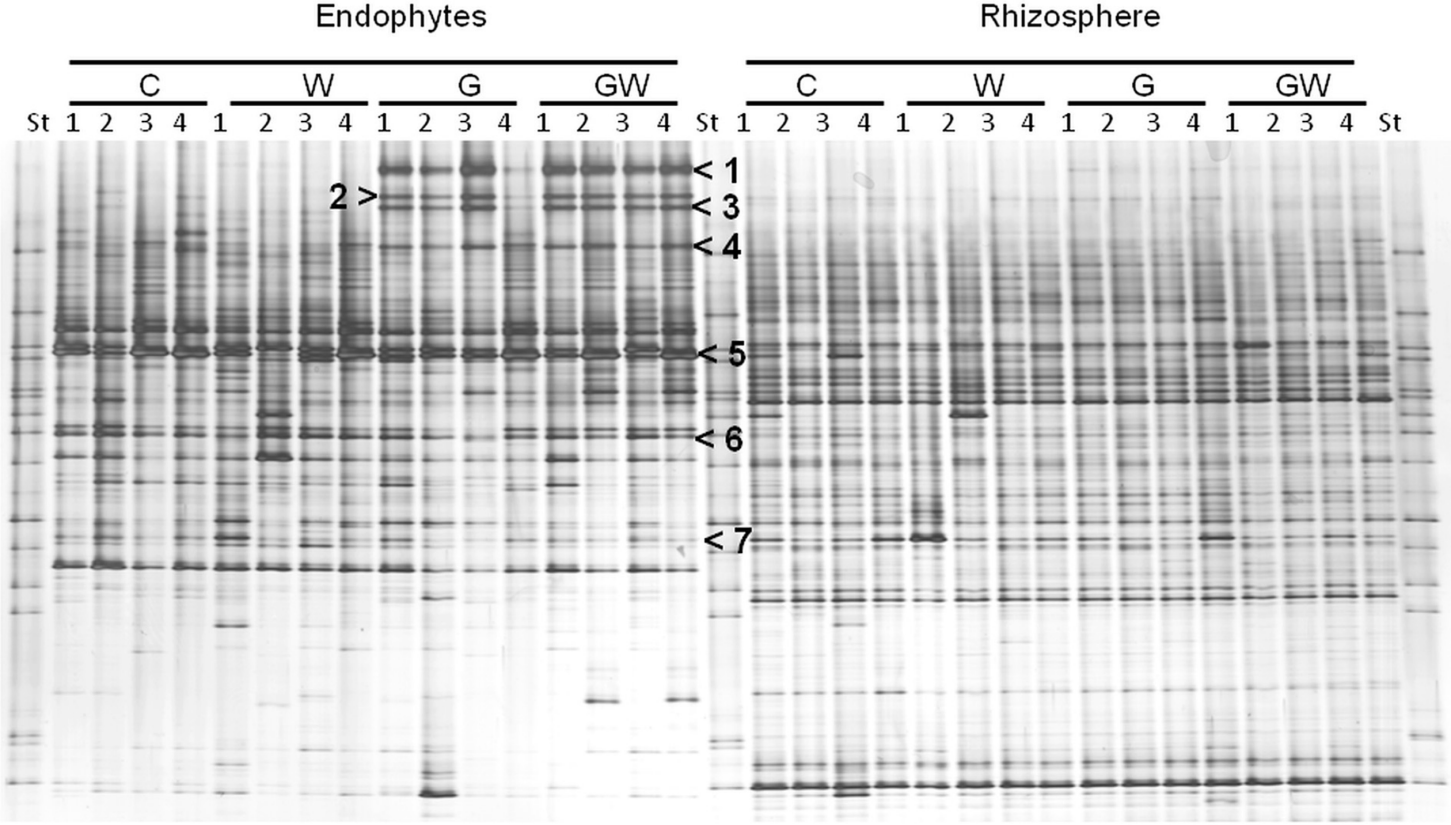
**DGGE fingerprints of ITS fragments showing the endorhiza and rhizosphere fungal communities of maize plants from the treatments C, W, G, and GW**. C, maize plant grown in Haplic Chernozem, natural source of different mycorrhizal species; W, maize plants characterized by 3 weeks root feeding by WCR larvae; G, maize plants with *GI* inoculum added before sowing; GW, maize plants mycorrhized by *GI* and characterized by 3 weeks WCR larval feeding on the roots; St, ITS standard. The fingerprinting was generated by separation of ITS fragments amplified from TC-DNA extracted from root and rhizosphere. Arrows indicate bands which were sequenced, except for band 4. Band 1, 2, and 3: *Glomus* sp.; band 5: *Microdochium bolleyi*; band 6: *Tetracladium* sp.; band 7: *Periconia macrospinosa*.

**Figure 3:**
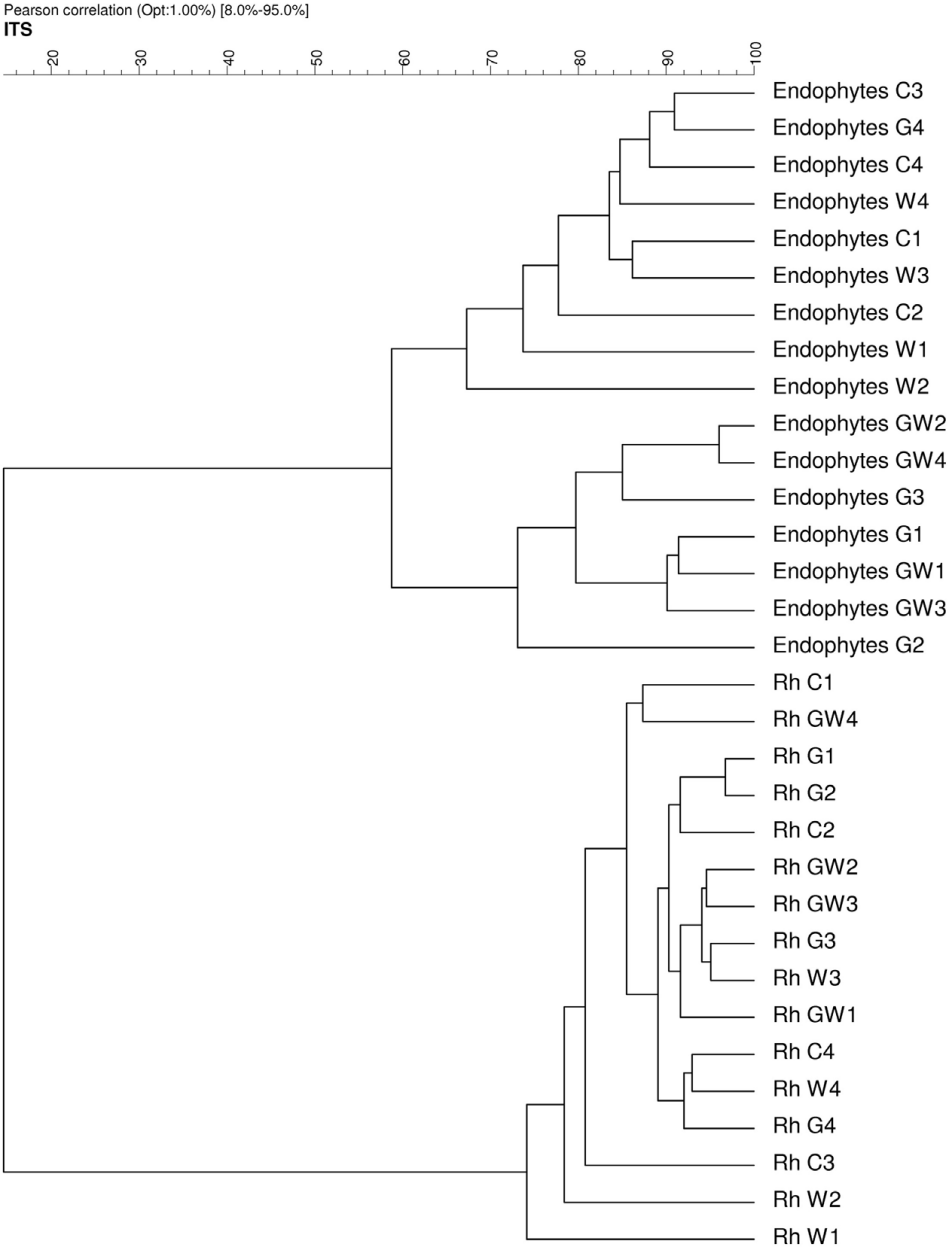
**Dendrogram constructed with the fungal communities fingerprints in the endorhiza and rhizosphere of maize reported in Figure 2**. The differences between the profiles are indicated by percentage of similarity. The dendrogram was based on the Pearson correlation indices and cluster analysis by the unweighted pair group method using arithmetic averages.

Comparative analysis of DGGE fingerprints of ITS fragments showed highly similar fungal community structure between the treatments C and W, and between the treatments G and GW in the endorhiza of maize. Four dominant differentiating bands appeared exclusively in the endorhiza fungal fingerprints of *GI* mycorrhized plants (bands 1, 2, 3, and 4, Figure 2). Cluster analysis of DGGE fingerprints of ITS fragments profiles showed that the treatments G and GW grouped together as well as the treatments C and W, with just one exception (Figure 3). However, differences (*P* = 0.03) in the fungal community composition observed between the treatments with and without *GI* (Table 3) indicated a clear effect of *GI* soil inoculation on the fungal populations in the endorhiza of maize. Differently, no effect of WCR larval feeding on the composition of the endorhiza fungal communities was observed.

**Table 3:** **Significant values (*P*-values) and *D*-values of pairwise comparisons between treatments (C, G, W, and GW) of fungal and bacterial communities fingerprints in the endorhiza and in the rhizosphere of KWS13 cultivar grown in Haplic Chernozem**.

	**Fungi**				**Bacteria**			
	**Endorhiza**		**Rhizosphere**		**Endorhiza**		**Rhizosphere**	
	***P***	***D***	***P***	***D***	***P***	***D***	***P***	***D***
C/W	0.3	0.8	0.5	0.3	**0.03**	8.2	**0.03**	7.8
C/G	**0.03**	8.7	0.06	2.9	**0.03**	5.2	**0.03**	7.1
C/GW	**0.03**	12.4	0.17	1.2	**0.03**	8.4	**0.03**	22.1
W/G	**0.03**	14.7	0.3	1.3	0.06	5.2	**0.03**	12.4
W/GW	**0.03**	23	0.08	1.3	0.1	5.1	**0.03**	13.4
G/GW	0.2	3.6	**0.03**	2.1	0.06	2.4	**0.03**	14.4

C, maize plant grown in Haplic Chernozem, natural source of different mycorrhizal species; W, maize plants characterized by 3 weeks root feeding of WCR larvae; G, maize plants mycorrhized by GI; GW, maize plants mycorrhized by GI and characterized by 3 weeks root feeding of WCR larvae. Values of P < 0.05 indicate significant differences between rhizosphere samples of different maize genotypes grown in the same soil type. Bold values show significant differences. Simulations: 10.000.

The DGGE fingerprints of the fungal communities in the maize rhizosphere showed high similarity among all treatments. A mixed cluster of samples from all treatments was obtained (Figure 3). Statistical analysis did not reveal significant differences between treatments with and without *GI*, indicating that *GI* soil inoculation did not affect the fungal communities in the rhizosphere. No significant differences were observed between treatments with and without WCR, except between G and GW with *P* = 0.03 and *D* = 2.1 (Table 3).

The BLAST analysis of the ITS-sequences obtained by cloning of bands 1, 2, and 3 (Figure 2) matched against the same type of *Glomus* sp., although with different percentage of similarity (96–100% identity) (accession no. JN36655–JN836661). No clones carrying an insert with the electrophoretic mobility of band 4 were obtained. Although this study focused on the identification of the four differentiating bands occurring only in *GI*-treated plants, other bands (bands 5, 6, and 7 in Figure 2) were also sequenced to obtain information on dominant fungal population in the endorhiza of maize. Band 5 was affiliated to *Microdochium bolleyi* with 99% sequence identity (accession no. JN836662 and JN8366623). Band 6 sequences showed 99% sequence identity with *Tetracladium* sp. (accession no. JN836664 and JN836665). The sequencing of band 7 revealed *Periconia macrospinosa* (98% sequence identity, accession no. JN836666).

### Bacterial communities in the endorhiza and rhizosphere of maize

In order to elucidate the interactions among WCR larval feeding, *GI* and bacterial populations inhabiting the rhizosphere and endorhiza of maize, a comparative analysis of DGGE fingerprints of 16S rRNA gene fragments was performed.

**Figure 4:**
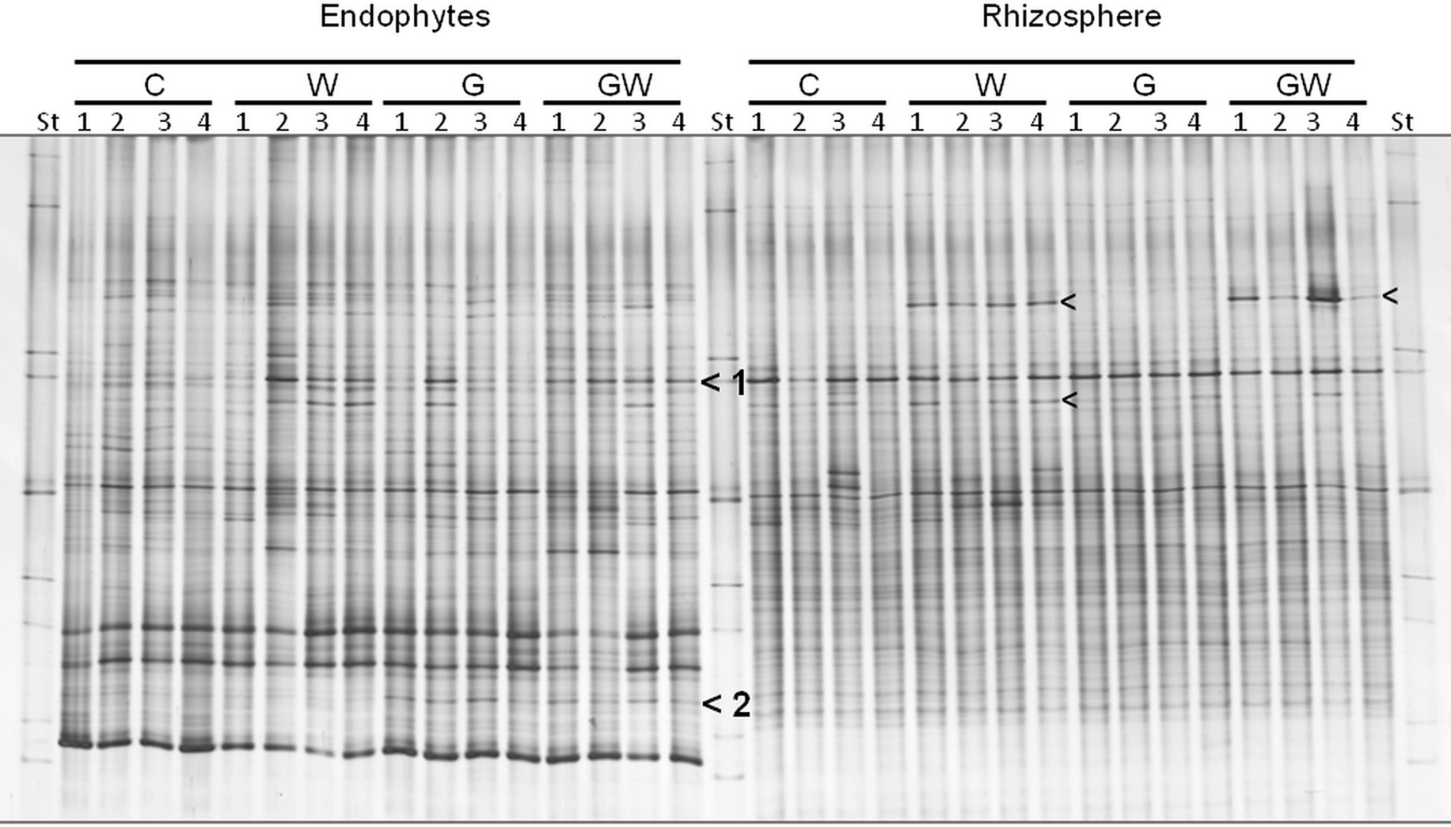
**DGGE fingerprints of 16S rRNA gene fragments showing the endorhiza and rhizosphere bacterial communities of maize plants from the treatments C, W, G, and GW**. C, maize plant grown in Haplic Chernozem, natural source of different mycorrhizal species; W, maize plants characterized by 3 weeks root feeding by WCR larvae; G, maize plants with *GI* inoculum added before sowing; GW, maize plants mycorrhized by *GI* and characterized by 3 weeks WCR larval feeding on the roots; St, ITS standard. The fingerprinting was generated by separation of bacterial 16S rRNA gene standard fragments amplified from TC-DNA extracted from root and rhizosphere. Arrows show treatment dependent bands.

The bacterial DGGE fingerprints in the maize endorhiza showed high variability among replicates. Differences in the relative abundance of two bacterial populations upon WCR larval feeding or of *GI*-soil inoculation were observed (bands 1 and 2, Figure 4). Statistical analysis based on the Pearson correlation indices revealed significant differences in the endorhiza bacterial composition between the treatment C and the treatments W, G and GW (*P* = 0.03) indicating a clear effect of both *GI*-soil inoculation and WCR larval feeding on the endorhiza bacteria in maize roots. Although a differentiating band (band 2, Figure 4) in the treatments with *GI*-soil inoculation was displayed, no significant differences were observed between the treatments W, G, and GW (Table 3).

**Figure 5:**
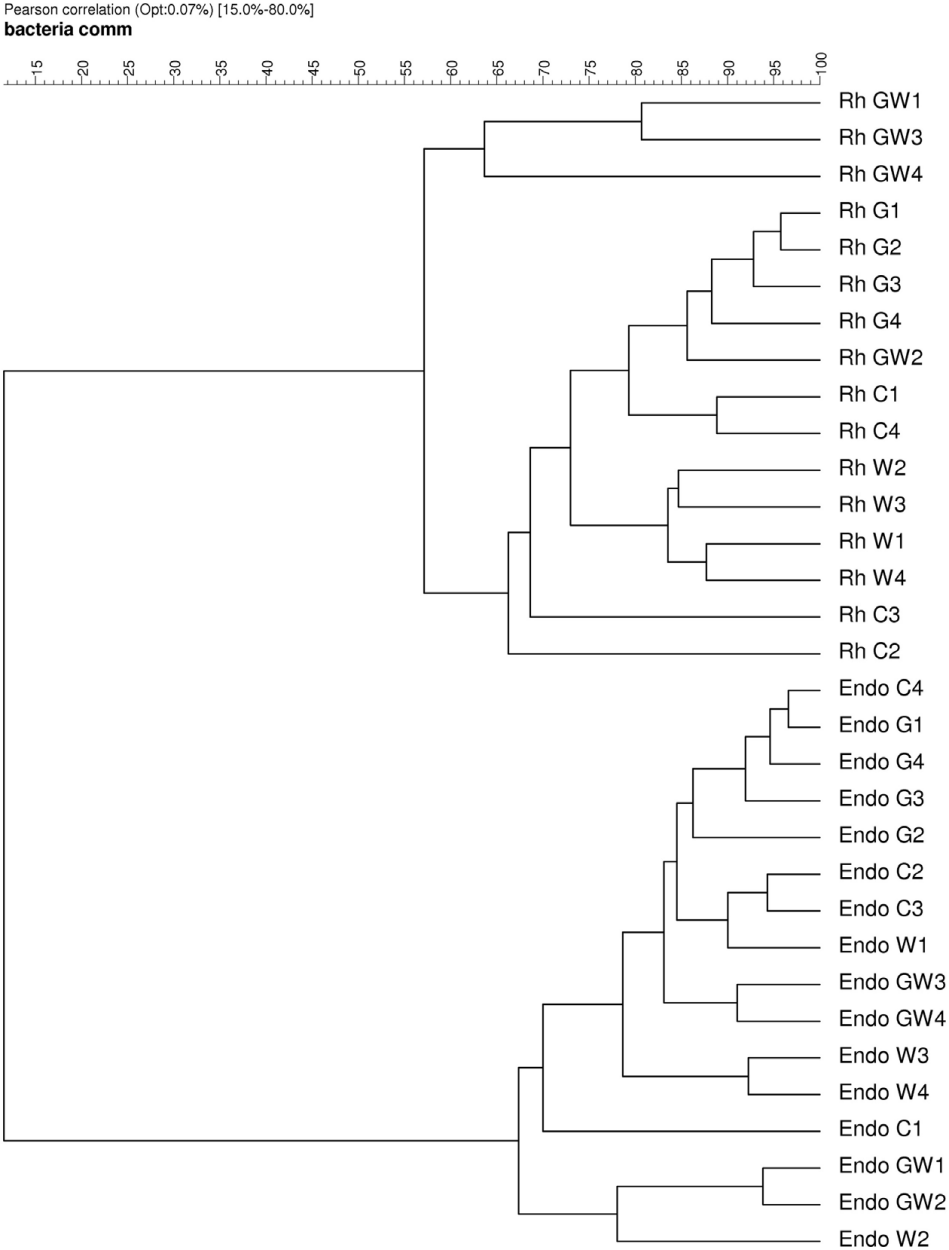
**Dendrogram constructed with the bacterial communities fingerprints in the endorhiza and rhizosphere of maize reported in Figure 4**. The differences between the profiles are indicated by percentage of similarity. The dendrogram was based on the Pearson correlation indices and cluster analysis by the unweighted pair group method using arithmetic averages.

The DGGE patterns of the bacterial communities in the rhizosphere of maize showed pronounced shifts due to the WCR larval feeding independently of the *GI*-soil inoculation, while no shifts were observed in response to *GI*-soil inoculation (Figure 4). No treatment dependent clustering was observed (Figure 5). However, statistical tests revealed significant differences between all of them with *D*-values of >7.1 (Table 3).

## Discussion

The present study provided new insights into the interaction among WCR larval feeding, *GI* and microorganisms living in the rhizosphere and in the endorhiza of maize. An inhibitory effect of the WCR larvae growth caused by the *GI* root mycorrhization was observed in the present study for the first time. Our findings are in agreement with Boucher (2001) who reported a reduction in head capsule diameter of emerging WCR beetles from *GI*-treated plants vs. control plants. According to the slow-growth-high-mortality hypothesis developed by Benrey and Denno (1997), the prolonged time of early larval instars renders WCR larvae more susceptible to predation by natural enemies. Therefore, *GI* can be proposed as a biocontrol microorganism for integrated pest management programs against WCR larval damages.

The mechanisms of the interaction between WCR larvae and *GI* remain unknown. However, the reduction of larval growth might be due to either a direct interaction between *GI* and WCR larvae or to plant-mediated interaction resulting, for instance, in root exudate changes. Maize secondary metabolites such as hydroxamic acids (Xie, 1991) or protease inhibitors and phenolic compounds (Karban and Baldwin, 1997) might have a toxic activity toward WCR larvae or limit the insect assimilation of plant nutrients and thus delay herbivore growth, respectively.

Several papers demonstrated the effect of the root exudation on shaping the rhizosphere and root-associated microbial communities and *vice versa* (Rettenmaier and Lingens, 1985; Bröckling et al., 2008; Berg and Smalla, 2009; Meier et al., 2012). The present study showed shifts in the indigenous endorhiza populations of AMF, fungi and bacteria in the *GI* treatments. Thus, we speculated that *GI* might act indirectly on the WCR larval growth via plant-mediated response to the presence of *GI* rather than directly affect specific compounds.

PCR-RFLP comparative analysis and sequencing of AMF-18S rRNA gene fragments amplified from DNA from soil and maize roots of plants grown in absence of *GI* showed significant differences in the AMF composition between soil and root samples, indicating a selective interaction between maize plants and the AMF populations naturally occurring in the soil. In particular, *G. mosseae*, uncultured *Glomus* species and *Scutellospora calospora* were positively selected by the plant most likely through the release of specific compounds (e.g., plant secreted proteins) mediating the process of signaling and recognition between compatible and incompatible plant-microbe interactions (De la Peña et al., 2008). PCR-RFLP analysis and sequencing of AMF-18S rRNA gene fragments in DNA from the roots of plants of treatments C and G revealed that *GI*-soil inoculation reduced the AMF richness in the maize roots to almost exclusively the RFLP type 11 identified by sequencing as *GI* itself. The dominance of *GI* in the roots indicated the preferential establishment of a mutualistic symbiosis between maize plants and *GI* rather than *G. mosseae*, uncultured *Glomus* species and *Scutellospora calospora*. As reviewed by Parniske (2008), the host preference reflects different fungal strategies and levels of functional compatibility.

Effects of *GI* inoculated into soil on the fungal and bacterial communities in the endorhiza and in the rhizosphere of maize were revealed. *GI* strongly affected the fungal community composition in particular in the endorhiza of maize (Figure 2). However, the effects were mainly caused by the appearance of bands in the fingerprints of endorhiza fungal communities in *GI*-treated plants that were all affiliated to *Glomus* sp. Thus, both qPCR and DGGE fingerprint data suggest that the inoculant belonged to the dominant endorhiza fungal populations associated with the maize. Some studies showed that ITS sequences were rarely recovered twice from a single spore (Lanfranco et al., 1999; Antoniolli et al., 2000), most likely due to the multiple and polymorphic genome of the AMF (Hijiri and Sanders, 2005). Furthermore, these data might indicate that the ITS region alone has a too low resolution power to differentiate AMF at the species level. *GI* inoculation affected significantly also the bacterial community composition in the endorhiza of maize, although to a much lesser extent (Figure 4). In the rhizosphere no clear differentiating bands on the DGGE fingerprints of fungal and bacterial communities were observed between the treatments with and without *GI*. However, permutation testing still indicated significant effects of *GI* treatment on the bacterial communities in the maize rhizosphere (Table 2). *GI* effects on the microbial communities in the rhizosphere and endorhiza of plants were reported also in other studies. Filion et al. (1999) showed that soluble substances released by the extraradical mycelium of *GI* induced differential growth of soil microorganisms. Marschner and Baumann (2003) showed that mycorrhizal colonization by *GI* changed the bacterial community structure in the soil and on the surface of maize roots.

WCR larval feeding on maize roots was found to strongly alter the bacterial community composition in the endorhiza and rhizosphere of maize in *GI*-treated and untreated plants. While WCR larval feeding did not affect the diversity of AMF and total endorhiza fungal communities in the maize endorhiza, the endorhiza bacterial communities of the treatments without *GI* were significantly affected. Interestingly, WCR feeding did not affect the bacterial communities in presence of *GI.* However, the absence of statistically significant differences could have been caused also by the high variability in the DGGE profiles between replicates. Confirming previous results by Dematheis et al. (2012), no change in the fungal community composition in the maize rhizosphere in response to WCR larval feeding was observed in the present study. However, larval feeding strongly affected bacterial communities in the rhizosphere of *GI*-treated and untreated maize plants. Thus WCR feeding influenced mainly the bacterial populations colonizing the rhizosphere and, to a lesser extent, those living in the endorhiza. One of the dominant bacterial populations occurring, upon larval feeding, in the maize rhizosphere of KWS13 maize, was recently shown by Dematheis et al. (2012) to share 100% sequence identity of the 16S rRNA gene with a phenol degrading *Acinetobacter calcoaceticus* strain. The identification of dominant bacterial populations responding to larval feeding in the maize endorhiza could be the subject of further investigations.

In conclusion, the present study provided new insights into the complex interaction among WCR larval feeding, *GI* and the microbial communities in both rhizosphere and endorhiza of maize. The most relevant result concerned the inhibitory effects of *GI* on the WCR larval development. The mechanisms of the interaction between WCR larvae and *GI* remain unknown, although our data suggested an indirect plant-mediated mechanism resulting in a shift of the microbial communities. However, our findings revealed for the first time a biocontrol activity of *GI* against WCR larvae which could be used in integrated pest management programs.

### Conflict of interest statement

The authors declare that the research was conducted in the absence of any commercial or financial relationships that could be construed as a potential conflict of interest.
